# Cytokines in agitated and non-agitated patients admitted to an acute psychiatric department: A cross-sectional study

**DOI:** 10.1371/journal.pone.0222242

**Published:** 2019-09-11

**Authors:** Jeanette Brun Larsen, Astrid Kamilla Stunes, Arne Vaaler, Solveig Klæbo Reitan

**Affiliations:** 1 St. Olav’s University Hospital, Department of Mental Health, Trondheim, Norway; 2 Norwegian University of Science and Technology, Faculty of Medicine and Health Science, Department of Mental Health, Trondheim, Norway; 3 Norwegian University of Science and Technology, Faculty of Medicine and Health Science, Department of Clinical and Molecular Medicine, Trondheim, Norway; 4 St. Olav’s University Hospital, Medical Clinic, Trondheim, Norway; Chiba Daigaku, JAPAN

## Abstract

**Background:**

Different psychiatric diagnostic groups have been reported to have cytokine levels deviating from healthy controls. In acute clinical settings however, the specific challenging symptoms and signs are more important than a diagnostic group. Thus, exploration of cytokines and immune activity and their role in specific symptoms is important. Reports in this field so far are sparse.

**Objective:**

In the present study, we aimed to examine the association between immune activity measured as levels of cytokines and agitation (independent of diagnostic group) in patients admitted to an acute psychiatric inpatient department.

**Methods:**

A total of 316 patients admitted to an acute psychiatric inpatient department were included. Thirty-nine patients with psychosis were subject to subgroup analyses. Agitation was assessed by the Positive and Negative Syndrome Scale, Excitement Component (PANSS-EC). Based on PANNS-EC patients were stratified into two groups: 67 agitated patients and 249 non-agitated patients. Serum concentrations of the following immune markers were measured: interleukin (IL) -1β, IL-4, IL-6, IL-10, tumor necrosis factor (TNF) -α, interferon (IFN) -γ and transforming growth factor (TGF) -β.

**Results:**

Serum levels of TNF-α were significantly higher in patients with agitation compared to those without, both when all patients were included in the analyses (p = 0.004) and in the psychosis group (p = 0.027). After correcting for multiple testing, only the findings in the total population remained significant.

**Conclusions:**

Our findings suggest an association between TNF-α and agitation in an acute psychiatric population. A similar trend was reproduced to the psychosis subgroup. This suggests that agitation might be an independent entity associated with cytokines across different diagnostic groups.

## Introduction

Different psychiatric diagnostic groups have been reported to have immune cytokine levels deviating from those seen in healthy controls [1, 2]. In clinical settings, the specific challenging symptoms and signs are more important than diagnostic groups. Thus, exploration of cytokines and immune activity and its role in specific symptoms and signs is important. In experiments, immune factors such as cytokines might influence the brain and mediate behavioral changes seen in psychiatric disorders [3, 4]. Clinical reports in this field so far are however sparse. Level of cytokines has been associated with severity of depressive symptoms in major depression and with negative symptoms in schizophrenia [5, 6].

Agitation is a challenging clinical sign. The term agitation may be defined as a syndrome of behaviors such as increased psychomotor activity, irritability, hostility, threatening gestures and lack of cooperativeness [7]. Acute agitation is particularly frequent in acute psychiatric services, and is associated with several psychiatric disorders, most commonly schizophrenia, drug intoxication and bipolar disorder [8, 9]. Agitation is causing suffering and may result in use of coercive means (involuntary medication, restraint, and seclusion) [10]. Untreated agitation may escalate to violence and adverse outcomes for patient, family, society and staff [10]. Thus, knowledge on mechanisms behind agitation is essential.

A few recent studies have shown a possible association between acute agitation in schizophrenia and the inflammatory marker C-reactive protein (CRP) [11, 12]. To our knowledge, only one previous study has investigated the association between cytokines and agitation, finding IL-17 and IL-23 to be associated with agitation [13]. However, this study was limited by a small study population and that it only included three different cytokines. It is also not known if the association between immunological markers (CRP or cytokines) and agitation is present in other diagnostic group than psychosis or schizophrenia.

There are also some studies on the association between aggression and cytokines. A study on inpatients diagnosed with schizophrenia, demonstrated a possible association between interferon (IFN) -γ and interleukin (IL) -10 and aggression [14]. However, the results are conflicting and another study found aggression to be associated with IL-17, IL-3, and transforming growth factor (TGF) -β [13]. Although aggression and agitation have several symptoms and traits in common, they are not completely the same. Therefore, we would state the association between cytokines and agitation is largely unknown.

The aim of the present study was to assess the levels of cytokines related to agitation in an acute inpatient setting. Levels of cytokines were compared between patients with or without symptoms of agitation measured with the Positive and Negative Syndrome Scale, Excitement Component (PANSS-EC). Agitation is known to be more frequent in certain patients groups, such as those with psychosis. Also, most previous studies have selected patients with psychosis when investigating the association between immune markers and agitation. As these reports are conflicting, separate analyses were done on the group non-affective psychoses. We hypothesized that the agitated patients would have higher levels of pro-inflammatory cytokines when compared to patients without agitation.

## Materials and methods

### Setting and participants

This was a cross-sectional study conducted in the acute psychiatric department of St. Olav’s University Hospital, Trondheim, Norway. All eligible inpatients acutely admitted between September 2011 and March 2012 were asked to participate. At the time of inclusion, the psychiatric department served a catchment area of 228.000 inhabitants (≥ 18 years). Of the total 654 admitted patients in the inclusion period, 382 (58.4%) patients gave written informed consent prior to inclusion. The study was approved by the regional committee for ethics (REC South East number 2011/137) and registered at ClinicalTrials.gov (NCT01415323). The study was conducted according to the Declaration of Helsinki.

### Inclusion and exclusion criteria

All patients evaluated to be able to give informed consent were asked to participate, independently of diagnostic group. Ability to give informed consent was evaluated by an experienced psychiatrist / clinical psychologist the first day after admission and in accordance with national and international regulations. Exclusion criteria were as follows: (1) chronic or ongoing infections, (2) comorbid autoimmune diseases, (3) CRP levels above 35 mg/L (level based on clinical experience), or (4) lack of patient consent. When patients had multiple admissions, we only included the first admission in our analyses.

### Diagnostic evaluation

Patients were diagnosed according to the International Classification of Diseases-10 (ICD-10) Criteria for Research [15]. The diagnoses were set in a consensus meeting always including at least two senior psychiatrists or clinical psychologist of whom at least one had personally examined the patient. All clinical data from patient files were available. For subgroup analyses, patients with non-affective psychosis (ICD-10 F20–29, psychosis group) and a group of all patients when excluding the psychosis group were studied.

### Assessments

Sociodemographic history, smoking status and the history of somatic diseases were recorded after an interview by a member of staff. Height and weight were measured in order to calculate body mass index (BMI, kg/m^2^). The patients were interviewed regarding substance use in addition to screening for drugs in the urine. In addition, participants were screened in a general medical examination and with routine blood tests, including CRP and leukocytes.

Agitation was assessed by experienced psychiatrists or clinical psychologist using the Positive and Negative Syndrome Scale, Excited Component (PANSS-EC). The PANSS-EC is a validated and commonly used scale assessing agitation in acute- and emergency psychiatry [16]. It is calculated as the sum of the following PANSS items: Excitement (P4), Hostility (P7), Tension (G4), Uncooperativeness (G8) and Poor impulse control (G14) giving a total scoring range 5 to 35. Clinically significant agitation is considered with PANSS-EC score ≥ 14 [17, 18]. For analyses, patients were divided into two groups: agitated patients (PANSS-EC ≥ 14) and non-agitated patients (PANSS-EC < 14).

### Immune markers

Blood samples were collected on 9 ml serum tubes with SiO2 without gel between 08:00 and 13:00 (median at 10:00) at the first working day after admission. Strict instructions regarding fasting were not given, though most patients would be fasting overnight. Samples were immediately cooled on ice, protected from daylight, and centrifuged within 30 minutes (15 min, 1500 g, 4°C). Serum samples were stored at -80°C in a registered Biobank (Biobank1, St. Olav’s University Hospital, Trondheim, Norway) until further analysis. The cytokines were chosen based on previous studies in our group as well as multiple reports from other groups in the field [19]. Also, the availability of commercial assays and general experience in the immune lab affected the cytokines chosen for analyses. An attempt was made to represent all the main assumed pathways of the immune system. The following parameters were analysed by multianalyte profiling Milliplex MAP assays: IL-1β, IL-4, IL-6, IL-10, TNF-α, and IFN-γ (Millipore Corporation, Billerica, MA, US). TGF-β was measured by a Bio-Plex Pro TGF-β Assay (Biorad Hercules, CA, US). All analyses were performed according to the manufacturers’ protocol. The range of detected values was as following: IL-1β: 0.06–198.72 pg/ml; IL-4: 0.92–286.01 pg/ml; IL-6: 0.10–576.42 pg/ml; IL-10 0.10-1125-33 pg/ml; TNF-α: 0.70–268.89 pg/ml; IFN-y: 0.04–1529.70 pg/ml; and TGF-β: 13.07–415.61 ng/ml. The number of samples and percentage under the detection limit was as follows: IL-1β 236 (74.2%), IL-4: 242 (76.1%), IL-6: 177 (55.7%), IL-10:177 (55.7%), TNF-α: 11 (3.5%), IFN-y: 68 (21.4%) and TGF-β: 0.

### Statistical analyses

Statistical analyses were performed using SPSS version 24.0 for Windows. The level of significance was set at p ≤ 0.05, and all analyses were two-tailed. Significant findings were adjusted for multiple testing with the Bonferroni correction (α/k where k = the seven tested cytokines giving α/k = 0.007). Data normality was assessed by using a Kolmogorov-Smirnov test. Only TGF-β was normally distributed and all the other cytokine values were skewed. Descriptive statistics were calculated by chi-square tests for categorical variables and student’s independent samples t-tests or Mann-Whitney U test (depending on distribution) for continuous variables. When comparing cytokine levels between two groups, such as agitated and non-agitated, we used a Mann-Whitney U test or a student’s independent samples t-test, depending on the distribution. Additionally, we examined the association between cytokines and possible confounders that are well known for influencing the immune system such as age, BMI and gender. The Spearman correlation coefficient was calculated for the relationship between cytokines, BMI and age. Patients were also stratified according to gender and cytokine levels were compared between female and male patients using a Mann-Whitney U test or a student’s independent samples t-test.

## Results

### Sociodemographic and clinical characteristics of the population

Of 382 patients initially included, 24 were excluded due to infection or autoimmune disease. For the remaining 358 patients, serum samples were available for cytokine analyses in 318 of these patients. Two of these patients were missing PANSS-EC scores and were therefore not included in the statistical analyses, leaving us with 316 patients in the analyses. The agitated group and the non-agitated group were similar in the demographic and clinical characteristics recorded in our study, including age, gender, smoking status and BMI (Table 1).

**Table 1:** Demographic and clinical parameters.

	All patients n = 316	Agitated patients n = 67	Non-agitated patients n = 249	p-value^a^
Age (years)	39.0 (26.2–49.0)	39.0 (27.0–56.0)	39.0 (26.0–49.0)	0.535^b^
Gender (female)	156 (49)	37 (55)	119 (48)	0.280^c^
Smoking	157 (50)^d^	38 (69)	119 (55)	0.056^c^
BMI (kg/m^2^)	24.4 (21.7–28.2)^e^	24.5 (22.0–27.6)	24.4 (21.6-28-4)	0.959^b^
Higher education (above high school)	48 (15)	7 (10)	41 (16)	0.223^c^
Unemployment (incl. sick leave)	224 (71)	53 (79)	171 (69)	0.104^c^
Alcohol use upon admission	87 (28)	19 (29)	68 (27)	0.811^c^
Substance abuse	64 (20)	15 (23)	49 (20)	0.584^c^

Data are presented as median with interquartile range or n (%)

^a^Comparison between agitated and non-agitated patients. Agitation was classified by Positive and Negative Syndrome Scale, Excited Component (PANSS-EC) score ≥ 14.

^b^Mann-Whitney U test

^c^Chi-square test

^d^Missing 44

^e^Missing 68

Abbreviations: BMI: body mass index, PANSS-EC: Positive and Negative Syndrome Scale, Excited Component, SD: standard deviation

The percentage of main ICD-10 diagnostic categories in the 316 patients were as follows: F32 depressive episode or F33 recurrent depressive disorder (n = 68, 21.5%)), F10-F19 Mental and behavioral disorders due to psychoactive substance use (n = 53, 16.8), F20-F29 schizophrenia, schizotypal and delusional disorders (n = 39, 12.3%)), F31 bipolar affective disorder (n = 42, 13,2%), F40-F48 neurotic, stress-related, and somatoform disorders (n = 32, 10,1%), and F60-F69 disorders of adult personality and behavior (n = 29, 9.2%).

### Comparisons of serum cytokine levels between agitated and non-agitated patients

**Fig 1:**
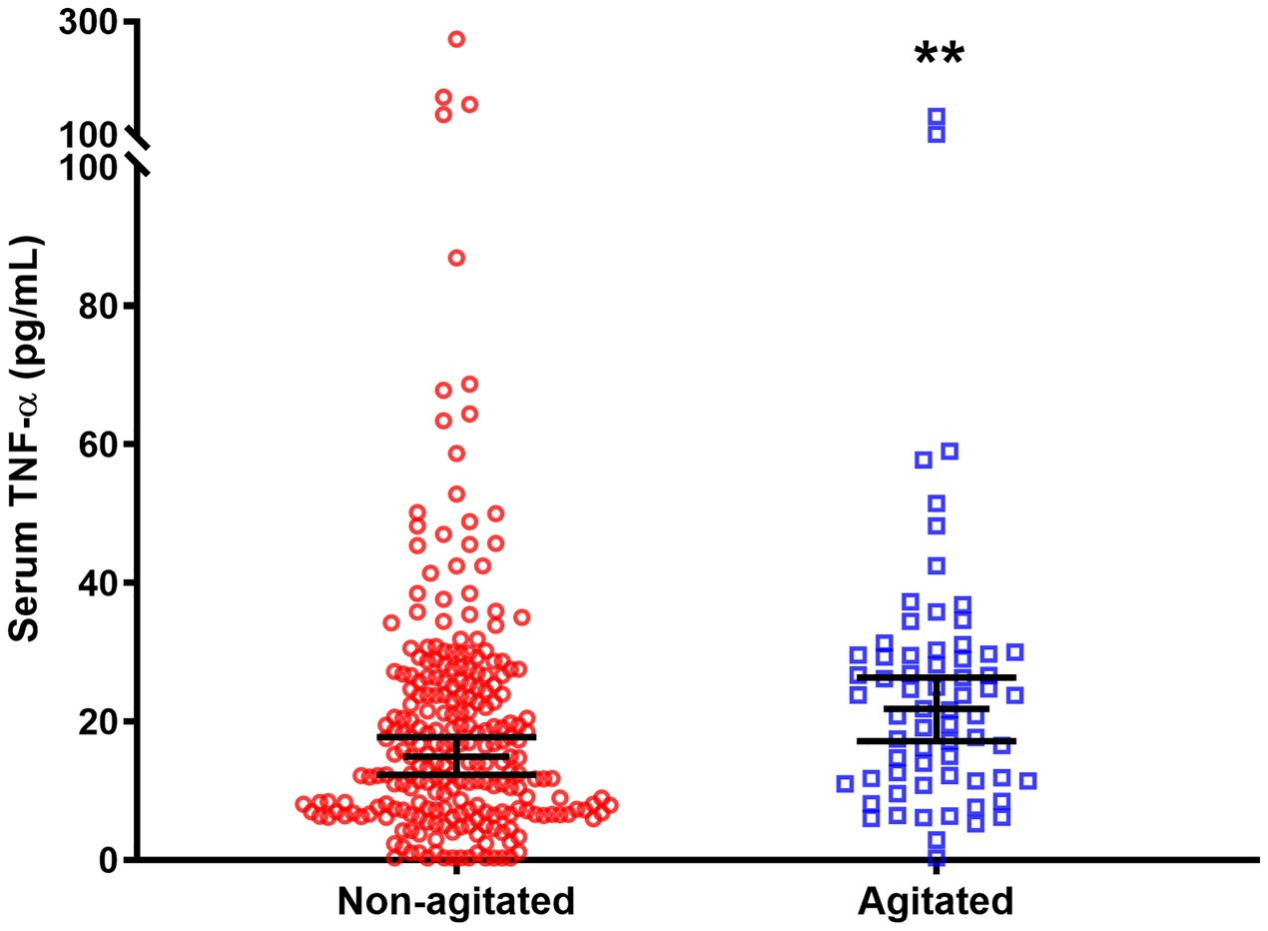
TNF-α levels in all acutely admitted patients. Serum TNF-α levels were significantly higher in agitated patients compared to non-agitated patients (21.87 ± 17.96 vs 14.94 ± 18.86 pg/mL). Data are expressed as median ± 95% confidence interval. **p = 0.004.

**Fig 2:**
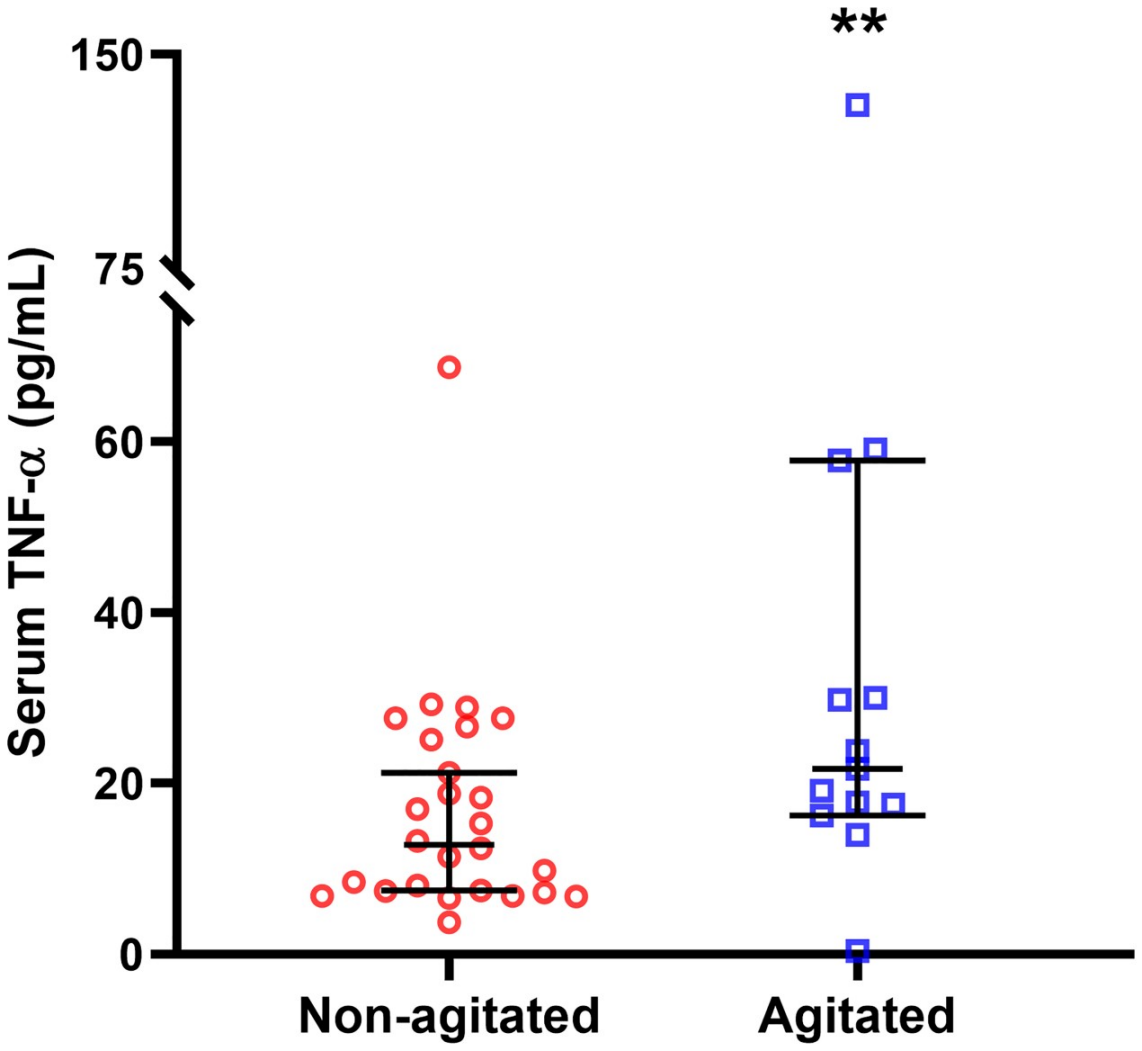
TNF-α levels in psychosis group. Agitated psychosis patients had significantly higher serum levels of TNF-α compared to non-agitated psychosis patients (21.71 ± 27.00 vs 12.84 ± 18.15 pg/ml). data are expressed as median ± 95% confidence interval. **p = 0.027.

**Fig 3:**
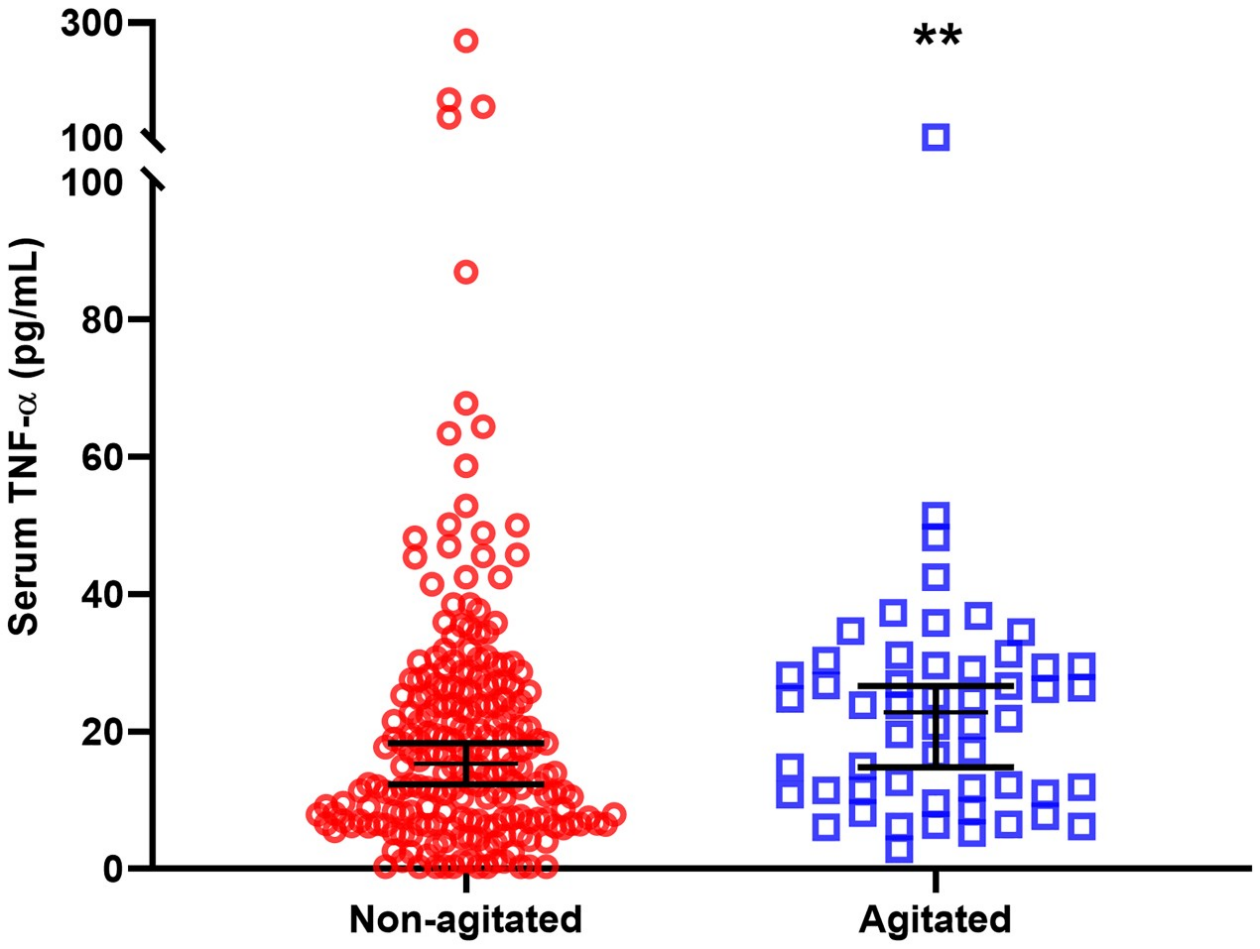
TNF-α levels in patients without psychosis. In a group of patients with other diagnosis than psychosis (psychosis group excluded), serum levels of TNF-α were significantly elevated in agitated patients compared to non-agitated patients (22.84 ± 18.23 vs 15.31 ± 19.22 pg/ml). Data are expressed as median ± 95% confidence interval. **p = 0.025.

When all 316 participants were included in the analyses, levels of TNF-α were significantly higher in agitated patients compared to non-agitated patients (21.87 ± 17.96 vs 14.94 ± 18.86, Mann-Whitney U = 6434.0, d = 0.16, p = 0.004) (Table 2 and Fig 1). This finding was replicated in the subgroup with psychosis, where agitated psychosis patients had significantly higher levels of TNF-α compared to non-agitated psychosis patients (21.71 ± 27.00 vs 12.84 ± 18.15, Mann-Whitney U = 95.0, d = 0.35, p = 0.027) (Fig 2). The finding of significantly higher levels of TNF-α in agitated patients when compared to non-agitated patients was also replicated in a subgroup analysis where the psychosis group was excluded (22.84 ± 18.23 vs 15.31 ± 19.22, Mann-Whitney U = 4841.0, d = 0.13, p = 0.025) (Fig 3). These subgroup findings were however not significant after the Bonferroni correction for multiple testing. In addition, there was a trend towards higher levels of IFN-γ in agitated psychosis patients when compared to non-agitated psychosis patients (20.90 ± 38.14 vs 3.49 ± 9.16, Mann-Whitney U = 105.0, p = 0.055), although not significant. No other differences in cytokine levels between the two groups reached the level of significance.

**Table 2:** Comparisons of cytokine levels based on the presence or absence of agitation ^a^. All data are presented as median with interquartile range.

	All patients			Psychosis group			Patients without psychosis		
	Agitated (n = 67)	Non-agitated (n = 249)	p-value	Agitated (n = 13)	Non-agitated (n = 26)	p-value	Agitated (n = 54)	Non-agitated (n = 223)	p-value
IL-1β (pg/mL)	0.03 (0.03–0.03)	0.03 (0.03–0.12)	0.724^b^	0.03 (0.03–0.03	0.03 (0.03–1.00)	0.415^b^	0.03 (0.03–0.14)	0.03 (0.03–0.09)	0.981^b^
IL-6 (pg/mL)	0.05 (0.05–45.38)	0.05 (0.05–17.58)	0.220^b^	0.05 (0.05–133.20)	5.66 (0.05–20.71)	0.547^b^	0.05 (0.05–31.43)	0.05 (0.05–15.65)	0.319^b^
TNF-α (pg/mL)	21.87 (11.83–29.79)	14.94 (7.26–26.12)	**0.004**^b^	21.71 (16.89–43.89)	12.84 (7.36–25.50)	**0.027**^b^	22.80 (11.33–29.56)	15.31 (7.13–26.35)	**0.025**^b^
IFN-γ (pg/mL)	7.05 (0.02–20.59)	5.12 (0.59–16.91)	0.907^b^	20.90 (1.48–39.62)	3.49 (0.08–9.24)	0.055^b^	3.62 (0.02–17.81)	5.20 (0.67–19.49)	0.477^b^
IL-10 (pg/mL)	0.05 (0.05–37.03)	0.05 (0.05–22.00)	0.423^b^	0.05 (0.05–85.73)	3.49 (0.05–54.48)	0.885^b^	0.05 (0.05–34.20)	0.05 (0.05–18.27)	0.405^b^
IL-4 (pg/mL)	0.46 (0.46–20.35)	0.46 (0.46–0.46)	0.110^b^	0.46 (0.46–39.47)	0.46 (0.46–7.52)	0.478^b^	0.46 (0.46–12.33)	0.46 (0.46–0.46)	0.193^b^
TGF-β (ng/mL)	78.41 (63.46–95.72)	80.47 (62.13–97.42)	0.924^c^	69.58 (60.31–95.21)	80.21 (63.57–93.51)	0.789^c^	78.68 (63.72–96.10)	80.47 (61.21–98.13)	0.687^c^

^a^Agitation was classified by Positive and Negative Syndrome Scale, Excited Component (PANSS-EC) score ≥ 14.

^b^Mann-Whitney U test

^c^Independent students’ samples t-test

Abbreviations: IL: interleukin, TNF: tumor necrosis factor, IFN: interferon, TGF: transforming growth factor.

#### Associations between cytokines and possible confounding factors

Serum levels of TNF-α did not differ significantly between smokers and non-smokers (14.93 ± 20.85 vs 16.81 ± 20.85, Mann-Whitney U = 8890.0, p = 0.696). Similar findings were reproduced for all the other measured cytokines. Age was negatively correlated with IFN-γ (rho = -0.150, p = 0.007), while no other significant associations between age and cytokines were found. In addition, there were no significant correlations between BMI and any of the measured cytokines.

**Fig 4:**
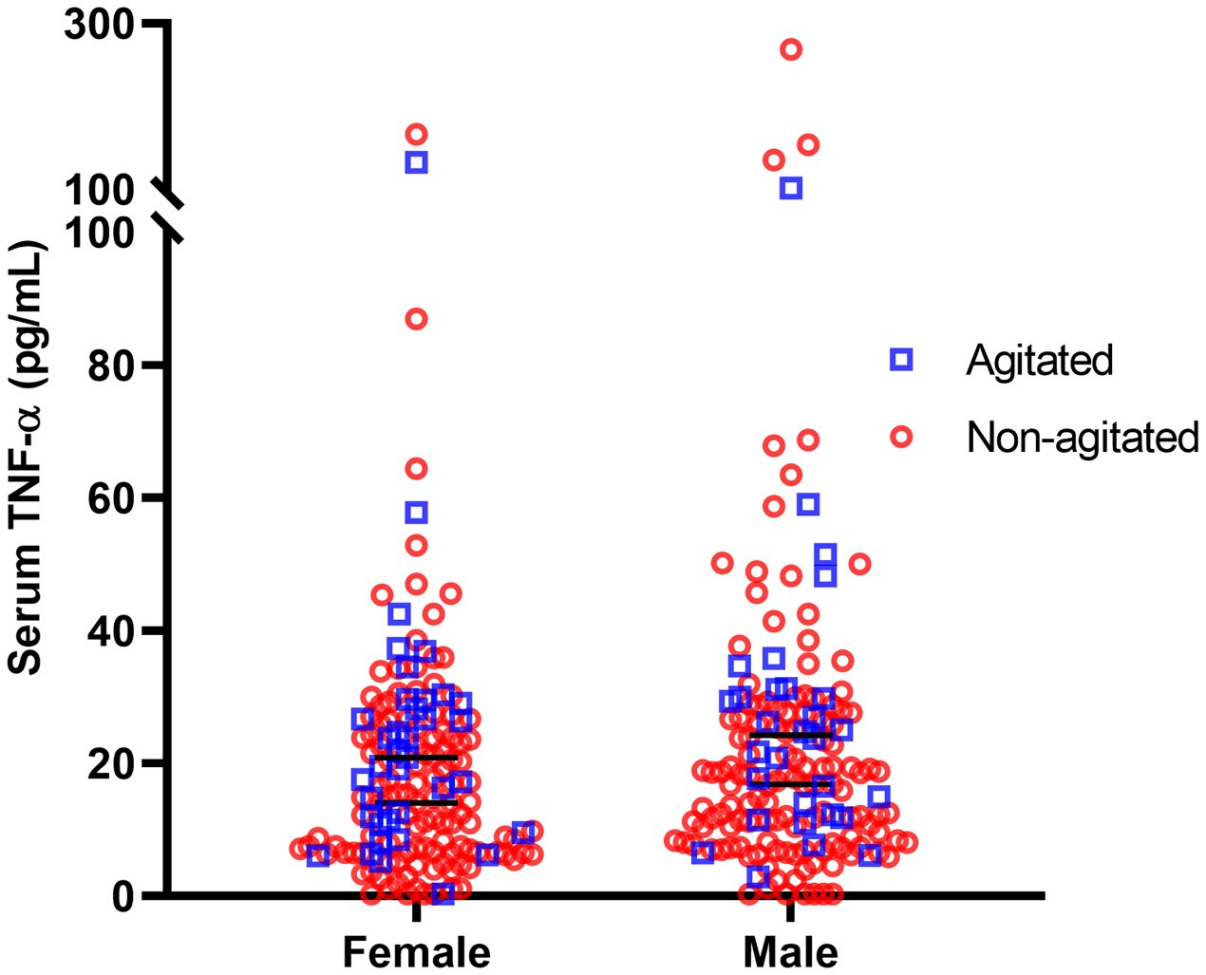
Serum TNF-α stratified by gender. No statistically significant differences were found in TNF-α levels between males and females (p = 0.143). TNF-α was significantly elevated in both male and female agitated patients when compared to non-agitated of the respective gender (female: 20.84 ± 18.17 vs 14.08 ± 18.23, p = 0.030, male: 24.27 ± 19.06 vs 16.82 ± 18.95, p = 0.045).

Stratification by gender showed that both female and male patients with agitation had higher levels of TNF-α than non-agitated patients of the respective gender (female: 20.84 ± 18.17 vs 14.08 ± 18.23, Mann-Whitney U = 1681.5, p = 0.030, male: 24.27 ± 19.06 vs 16.82 ± 18.95, Mann-Whitney U = 1492.0, p = 0.045). In addition, TNF-α levels did not differ significantly between females and males (15.13 ± 19.79 vs 17.75 ± 19.31, Mann-Whitney U = 11440.0, p = 0.143) (Fig 4). Levels of IL-6 (Mann-Whitney U = 10864.0, p = 0.017) and IL-10 (Mann-Whitney U = 10415.5, p = 0.003) were significantly higher in male subjects. No other gender differences in cytokine levels were detected.

## Discussion

### Summary of results

The present study indicates that serum TNF-α is increased in acutely agitated psychiatric inpatients when agitation is measured by PANSS-EC. This finding was present in both the general acute psychiatric population, within the diagnostic subgroup of non-affective psychosis and when the subgroup of non-affective psychosis was excluded from the population. However, the finding only remained significant in the general population when correcting for multiple testing. Serum levels of TNF-α were not affected by gender, age, smoking status or BMI. In agitated patients with psychosis, there was a trend towards increased IFN-γ when compared to non-agitated patients with psychosis. Increased IFN-γ was related to younger age but not to other potential confounding factors.

### Elevated TNF and agitation

Previous studies on patients with schizophrenia have shown an association between increased PANSS-EC and the inflammatory marker CRP [11, 12]. As CRP is produced by the liver when stimulated by cytokines such as TNF-α, these studies are in line with our finding of increased TNF-α levels in agitated patients with schizophrenia as well as in the general acute psychiatric population. Hostility is one of several symptoms seen in the syndrome of agitation. Hostility is also one of the items scored in the PANSS-EC. Therefore, the association of TNF-α and IFN-γ with hostility reported in healthy subjects are might also in line with our results [20].

Li et al. found that agitation measured by PANSS-EC was correlated with the cytokines IL-17, IL-23 and TGF-β in patients with schizophrenia [13]. This finding is not in line with our results, as we found agitation to be associated with TNF-α. However, Li et al did not measure TNF-α and IFN-γ, and we did not measure IL-17 or IL-23. It is possible that our findings would be more in line if measuring the same cytokines.

A few studies have also investigated the association between aggression and cytokines. As aggression and agitation are two overlapping clinical signs with several symptoms in common, these findings are also relevant to our study. Previously, levels of IFN-γ, IL-10 and TGF-β have been reported to be associated with aggression in schizophrenia patients [13, 14]. In line with this, we found a trend towards an association between IFN-γ and agitation in the sub-group non-affective psychoses. Interestingly, this was not seen in our total population, indicating that it may be a phenomenon specific for non-affective psychoses. The phenomenon should be further explored in a larger study and comparing with other diagnostic groups. For IL-10 and TGF-β we could not confirm the findings previously reported in a single study. It is also possible that patients with agitations have slightly different changes in the immune system than those seen in patients with aggression. However, only two studies is too limited to conclude and the phenomena should be further explored.

Some of these cytokines may be described to suppress or enhance each other, a phenomenon that could be part of the discussion. However, the patterns of interaction between different cytokines in immunology, as well as in neurophysiology, may have to be revised. Also, as stated decades ago the effect of cytokines on behavior may be context-dependent [21]. Thus, we do not comment further on in this article.

### Potential confounders

The possible confounders age, gender, BMI and smoking, affected few cytokines in our study. This is in contrast to the established consideration of high BMI and obesity as a driver of chronic low-grade inflammatory state [22]. This knowledge is mainly yielded from animal studies, epidemiological studies and other populations without a psychiatric comorbidity [23]. However, in a study from an acute psychiatric population elevation of inflammatory markers, independently of BMI, has been reported [24].

Previous studies in acute psychiatry have shown that high levels of inflammatory markers might also be independent of smoking, age and gender [12, 25]. The same finding was reproduced in a population of depressed patients where the association between cytokines and anhedonia was independent of gender [26]. It is possible that the association between possible confounders and inflammatory markers is overridden by inflammation related to the acute psychiatric state in our population. Also, this study is on cytokines, not CRP and changes in TNF-α might be less related to BMI than CRP.

Medications is another potential confounder. The effect of clozapine and other neuroleptics as well as other psychoactive medications on the immune system is well known [27]. A slight effect of medications on levels of cytokines in psychiatric populations has been reported, as reviewed by Goldsmith et al [2]. Unfortunately, we did not record the use of medications in this study. The acute setting with blood samples withdrawn the first day after admission reduces the relevance of this issue, as most patients would be medicated later. Also, assessment of medications used immediately previous to admission is difficult in this clinical setting; patients are psychiatrically very ill and may not be able to actually report use of medications correctly. In addition, many patients are admitted due to exacerbation after discontinuation of medications. Other patients have not started medications when acutely admitted, maybe for the first time. Use of psychoactive substances is a similar issue.

Independently of the cause of the altered cytokines and the potential confounders, we suggest that the findings may be of clinical relevance. The purpose of our study was to reveal factors that may be a cause of agitation, and consequently a target for prevention and relief of agitation. No matter whether the potentially altered cytokines—that potentially cause agitation—are an internal trait of the psychiatric disorder or a consequence of known or unknown confounding factors (like medications) they may be a target for clinical improvement. Clinical relief of symptoms and signs is the main goal of clinical research. We fully agree that any confounding factor should be explored. However, at the same time the clinical relevance must be kept in mind. Thus, we suggest our findings are interesting also independently of confounding factors. In this context, it may even be argued that high CRP should not have led to exclusion.

### Limitations and strengths

There are several limitations to the current study. The study population in the psychosis group is relatively small. However, the population is rather high when including all patients and when comparing the number with other studies in acute psychiatry.

We do not have a healthy control group that could extend the interpretation of the study. We did however aim to investigate characteristics of agitation and thus comparing with non-agitated patients may be more suitable than comparing with (non-agitated) healthy controls.

No data on medication was available and we did not control for substance abuse in our analyses. Theoretically, medications as well as substances affecting agitation also might affect the immune system. Substance abuse is also a possible confounder that we did not adjust for in our analyses. However, we did not find any significant differences in substance abuse between the agitated and non-agitated group. The cross-sectional design is another limitation, making it impossible to draw any conclusion on the causal connection between inflammation and agitation.

All blood samples were drawn during the first 24 hours of admittance to a closed inpatient ward, when the symptoms were most prominent and patients acutely ill. We used a validated and well-known tool for assessing the syndrome of agitation, which simplifies the comparability with other studies in the field. Furthermore, the psychiatric department recruiting the study participants is the only inpatients service in the catchment area, reducing the effect of socioeconomic differences.

## Conclusions

The present study indicates that there is an association between the syndrome of agitation and TNF-α in an acute psychiatric population. A similar trend was reproduced to the psychosis subgroup. The findings need to be replicated, but may lead to therapeutic interventions in the future.

## Supporting information

S1 File Full dataset. (SAV)
